# Utilization of dental care services among adult Indian population: A meta-analysis of evidence from 2011–2022

**DOI:** 10.34172/hpp.2022.42

**Published:** 2022-12-31

**Authors:** Rounik Talukdar, Diplina Barman, Vallabh Thakkar, Suman Kanungo

**Affiliations:** ^1^Division of Epidemiology, ICMR-National Institute of Cholera and Enteric Diseases, West Bengal, India; ^2^Jan Swasthya Sahyog, Chhattisgarh, India

**Keywords:** Oral health, Dental care, Dental health services, Utilization, Meta-analysis, India

## Abstract

**Background:** This study aimed to generate a pooled national estimate on dental health care services utilization by the adult population in India from any public or private facility in an effort to highlight the demand and usage for oral health care.

**Methods:** In this meta-analysis, PubMed, ScienceDirect, DOAJ, and Google Scholar were searched using a search strategy that combined MeSH headings and keywords (e.g., "Oral Health", "Dental Health Services", utilization, India, etc.) for articles on dental utilization among Indian adults, published between January 2011 and June 2022. Study quality was assessed using the NIH Quality assessment tool, and a random-effects inverse-variance method was used for pulling utilization proportions. Meta-regression and sub-group analyses were conducted to identify the sources of heterogeneity. Heterogeneity is reported as I^2^. To examine publication bias, the funnel plot, egger’s test, and trim-and-fill analysis were performed.

**Results:** From 4012 identified articles, 21 were eligible for inclusion. The pooled dental care utilization amongst Indian adults were found to be 23.96% (confidence interval [CI]: 16.81%– 31.11%, *P*<0.001, I^2^=98.93%), and the highest estimate was in South Zone (30.02%, CI: 19.14–40.90, *P*<0.01, I^2^=98.63%). Visual inspection of the funnel plot revealed the presence of publication bias (egger’s *P* value 0.02). A mild decrease in utilization estimate was noted through trim and fill analysis (adjusted estimate 17.65%, CI: 8.97–26.33, *P*=0.03). No significant subgroup effect was found for the variables study zone and conduction years (*P* value: 0.09 & 0.34 respectively).

**Conclusion:** Future studies should be undertaken to focus on the demand and supply of oral health care services since an evidential gap has been identified due to the uneven distribution of studies available from various regions of India. The heterogeneity can be attributed to the diverse socioeconomic, literacy, and inherent health system performance status.

## Introduction

 Even though universal health coverage’s (UHC’s) central tenet of equal, accessible quality healthcare for all has spurred considerable improvement across all health-related fields, oral healthcare has seldom been overlooked.^1,2^Global Burden of Disease 2019 estimates that around 3.5 billion people worldwide are affected by any form of dental condition, among which the most prevalent are untreated dental caries and periodontal conditions.^3,4^ Most Dental diseases are chronic and progressive, which mimics the patterns of most other noncommunicable diseases.^5^ Simpson et al in a systematic review reported that treating periodontal diseases was associated with better metabolic control in patients suffering from type 2 diabetes.^6^ Moreover, studies have shown a direct correlation between malnutrition and unresolved dental disease.^7^ These pose a greater concern on the public health canvas as the status of oral health is linked with the country’s socio-economic, and commercial developments.^3^

 Even though India’s National Oral Health Program (NOHP) has been existing since 2014, oral health is still tainted with low levels of individual awareness, inadequate infrastructure and resources, lack of relevant dental research, etc.^8,9^ Batra et al^8^ reported a prevalence of 79.2% and 84.7% dental caries amongst Indian adults of aged 35–44 & 65–74 years respectively. Further, India caters to the burden of having 1/3rd of all oral cancer cases in the globe.^10^ Besides the disease burden and availability of dental services at different strata, the utilization of these services has not been much looked upon.

 Therefore, the need of the hour is to strengthen our existing oral health policy. Real-world data is needed for any policy reform to happen. Data on the burden of the problem, and healthcare service utilization (demand and supply) helps decision makers to allot or reorient resources according to the need of the population. There is a dearth of evidence gauging pan-India oral healthcare utilization. Gellman defined health care utilization as the usage of health care for diagnosis, treatment, promotion of healthy living, and learning about one’s health state. Estimation of oral healthcare utilization would not only provide data on demand and supply of services but also will act as a proxy indicator for the persistence of various oral disabilities in different regions of the country.^11,12^ Hence we conducted this meta-analysis as a pioneering step to curate evidence available throughout India regarding public or private/any type of dental services utilization amongst the adult population to attain a pooled pan India estimate.

## Materials and Methods

 The meta-analysis was registered in the Open Science Framework database after its conceptualization stage bearing preregistration id. doi.org/10.17605/OSF.IO/7VAPW. Any deviations from the primary registrations have been described. We reported our findings adhering to the Preferred Reporting Items for Systematic Reviews and Meta-Analyses (PRISMA) statement.^13^ The PRISMA checklist has been provided in Supplementary file 1.

###  Strategies for search

 A systematic search was made amongst four digital databases including: PubMed, ScienceDirect, DOAJ, & Google Scholar on June 30, 2022, to curate evidence published relevant to our objective between January 2011 to June 2022. We did not include studies that have been published beyond 2011, to make our review relevant to today’s changing healthcare scenario. For the first three databases, our search was as broad as possible. The central theme of our search strategy has been described below:

 ((“Oral Health”[Mesh]) AND (“Dental Health Services”[Mesh]) OR (“dental”[Title/Abstract]) OR (“oral health”[Title/Abstract]) OR (“dentist”[Title/Abstract])) AND ((“utilization”[Title/Abstract]) OR (“seeking”[Title/Abstract]) OR (“provision” [Title/Abstract]) OR (“use of services”[Title/Abstract])) AND (“India”[Title/Abstract])

 Search strings were manually translated according to the search style of other individual digital databases used in this study. To limit searches in Google scholar in relevance to our objective we used “allintitle” in the advanced search filter.

###  Selection criteria

 We structured our selection criteria following the CoCoPop approach, which has been advocated as a preferable method in systematic review and metanalysis of prevalence/proportions.^14^ The framework was described as: Condition (dental care utilization), Context (India/received dental care services from public/private any of the facilities as per definition of dental utilization*), Population (adult Indian population).

 Studies adhering to the below inclusion criteria were considered: 1. Observational studies, 2. Community-based prospective/cross-sectional studies, 3. Studies bearing adequate data in the public domain to extract the numerator and denominator for calculation of dental utilization*/ proportion. 4. Studies including participants in the age group of 18 years and above or, studies from which data on dental utilization by the aforementioned age group could be extracted. 5. Studies conducted on the Indian population alongside reporting dental utilization as an outcome. 6. As the review concentrates on India, studies published only in the English language were included, which holds the status of the associate official language of India.

 Studies were excluded based on the following criteria: 1. Any study based on secondary data, 2. Unpublished studies and grey literature were not included in our study because of expected insufficiency in reporting the data.

 Since parental behaviour significantly influences a family’s dental health care utilization patterns, studies involving children, school-age children, and teenage participants under the age of 18 have been excluded. In addition, we wanted to identify the utilization patterns of individuals in the productive age group (15–64 years) and elders.^15,16^

 *Dental utilization was defined in this review as:

1. Dental care services at any public/ private institutions availed due to any reason at least once by the participants in the last 1 year.
2. Studies that have reported dental visits as ‘regular’ defined as twice in a year/ once in six months.

The American Dental Association recommends routine dental check-ups at least once a year, moreover a Cochrane review by Fee et al has extensively summarized the evidence in support of routine dental check-ups every six months or once a year. These facts serve as a basis for the definition of dental utilization used in this study.^17,18^

###  Study selection

 Title screening of the studies gathered using the search strategy from all the databases in the first hit was conducted to identify studies that fit the inclusion criteria by two reviewers (VT, & DB) independently. After title screening selected studies were imported to Zotero (version 5.0) for identifying duplicates. Any disagreement between the two reviewers was resolved through discussion amongst VT, DB, and another reviewer RT. Next followed the abstract screening of the identified studies. Full texts were retrieved for the selected abstracts by RT, DB, VT & SK and were subsequently thoroughly reviewed for eligibility and sufficiency of data. After excluding studies with a mismatch in methodology and set criteria & definitions used for outcome in our study, a list of final selections was obtained.

###  Data extraction

 A data extraction form was created in the online google sheets platform to dynamically verify the data amongst reviewers. The spreadsheet included the following information: 1. Unique identifier for each study, 2. First author details, 3. Publication year, 4. Study type, 5. Study settings (urban/ rural), 6. Study zone amongst six geographic zones of India, 7. The population involved, 8. Sample size, 9. The age group & mean age of the population included, 10. Number of participants utilized dental care services according to our criteria, and 11. Any other major theme/ outcome relevant to the study objective. Data extraction was conducted by VT & DB and was subsequently reviewed by SK.

###  Quality assessment

 All the full texts, finally listed for inclusion, were assessed using NIH Quality Assessment Tool for Observational Cohort and Cross-Sectional Studies. The tool contains 14 questions examining methodological rigor and the quality of the studies overall. In this review, eight questions relevant to cross-sectional studies were assessed, whereas six questions addressing chiefly observational cohorts were excluded. Each question with a satisfiable answer was awarded a score of 1, otherwise zero. Questions that were not relevant to the studies included in this review were not counted in the denominator for calculating the individual scores. Two reviewers RT, and DB assessed the quality of studies independently which was further discussed in consensus with SK for resolving any disagreement. The complete NIH assessment tool can be found on the nhlbi.nih.gov website.^19^

###  Data synthesis and analysis

 The main outcome of this review was dental care services utilization at any type of service point (public/ private) among the adult population in India, which was described as a proportion with lower and upper confidence limits.

 R Studio (version 4.1.3, 2022; The R Foundation for Statistical Computing, Vienna, Austria) was used for conducting the meta-analysis.^20^ A dedicated command package for meta-analysis of single proportions: ‘metaprop’ (Rdocumentation.org) was used. As well as incorporating the Freeman-Tukey double arcsine proportional transformation, it enables the construction of 95% confidence intervals (CIs) using appropriate statistical approach. The application also enables the binomial distribution to be used to model the within-study variability.^21^ Extracted data were utilized to calculate the standard error (SE) of the proportions of the population utilized dental services as per defined criteria in this study. The formula used is depicted below:

 SE = √(*P* × (1–*P*) ∕ n), *P* = proportions, *n* = population size.^22^

 For pulling proportions a random effect inverse variance method was used which involves the calculation of weighted average using SEs, to be supplied to the data frame for “metaprop”^22^ package in R Studio (version 4.1.3, 2022; The R Foundation for Statistical Computing, Vienna, Austria).^20^ The justification for using a random effect model is based on the fact that a considerable amount of study level heterogeneity in terms of research population, location, and methodology was anticipated during the selection and finalization of the studies. Furthermore, in their book “*Introduction to Meta-Analysis*,” Borenstein et al argued for selecting a random or fixed effect model based on conceptual grounds rather than Q statistics.^23^The pooled estimate was reported as a proportion with a 95% CI and graphical representation in the form of forest plot was done as required. I^2^ statistics were performed to assess heterogeneity. I^2^ statistics for heterogeneity interpretation was as follows: < 25% - mild, 25-75% - moderate, and > 75% - high.^24^To evaluate heterogeneity, sub-group analyses were performed based on the year of study, and study zones. Univariate meta-regression was conducted to understand the probable reason for heterogeneity for the following variables: study zones, study settings, year of study, and gender of participating population.

 Sensitivity analysis was done with the removal of outliers to assess the variation in the pooled estimates of the outcome variable. Possible outlier studies were identified in terms of reporting of dental care utilization using Tukey’s (1977) method. A possible outlier is defined as: values that fall outside the range of 1.5 times IQR below quartile one (Q1) or 1.5 times IQR above quartile three (Q3).^25^

 Assessment of publication bias was done using a funnel plot, and asymmetry of the plot was tested using Begg’s and Egger’s test where *P* value of ≤ 0.05 was considered statistically significant.^26^

 Further, trim and fill analyses were performed to determine the reliability of our estimate by calculating the adjusted pooled prevalence.^27,28^The test achieves this by detecting potential missing studies due to publication bias and attempts to recalculate the pooled prevalence by taking those missing studies into account thereby balancing the funnel plot.^29^The command “trimfill.rma.uni” which is inherently loaded in “metafor” R package was utilized for the analysis, further a funnel plot with inputted missing studies and adjusted utilization estimate was constructed.^30^

## Results

###  Screening and selection of studies

**Figure 1:**
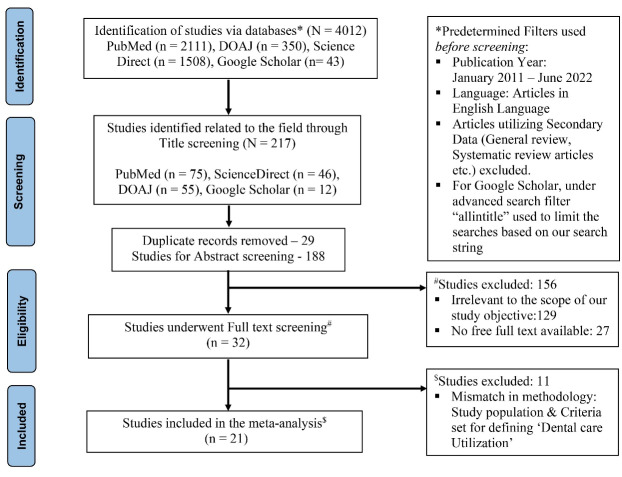
Flowchart of the process of searching and selecting studies based on PRISMA guidelines

 A total of 4012 studies were identified on four databases through the first hit, (PubMed–2111, ScienceDirect–1508, DOAJ–350, Google Scholar–43). Out of these 4012 studies, 217 were identified as relevant to the broader theme of this review & meta-analysis through title screening, further 29 studies were found to be duplicates and were removed. Rest 188 studies had undergone abstract screening amongst which 156 studies were excluded for either being irrelevant to our objective or due to unavailability of free full texts. The full-text screening was conducted for 32 articles, of which 11 were excluded based on methodological mismatch from our inclusion criteria. Finally, 21 articles were included in this review. A complete flowchart following PRISMA guidelines has been depicted in Figure 1.

###  Characteristics of the studies selected

 Amongst the 21 studies included, eleven studies were from the year 2017–2022 group in terms of the year of the study conduct, whereas the other ten belonged to the 2011–2016 group. The median sample size in the involved studies was 427 with a minimum being 35 and a maximum being 5476. The interquartile range for sampling size was 294.5 (Quartile 1) - 858.5 (Quartile 3). The study done by Gupta et al^31^ included 5476 participants which is 3.74 standard deviations away from the mean and was considered a definite outlier. Whereas the study by Pradeep et al^32^ (2934 participants) was 1.7 standard deviations away from the mean sample size. Most of the studies (11 out of 21) included in this review were from the south zone of the country. Out of 21 studies there were 5 from urban and 7 studies from rural areas respectively, whereas the rest enrolled participants from both (urban/rural) settings. Table 1 describes the complete details of the studies.

**Table 1:** Characteristics of studies included in review

**First author, Year, References**	**Study type**	**Study setting**	**Study Zone**	**Age range included (in years)**	**Place, State**	**Sample size**
Bhatt, 2018^33^	Cross-sectional study	Urban	South	18-21	Mangalore, Karnataka	575
Nagarjuna, 2016^34^	Cross-sectional study	Both	South	≥ 20	Nellore, Andhra Pradesh	600
Reddy, 2016^35^	Cross-sectional study	Urban	South	≥ 20	Hyderabad, Telangana	1017
Salunke, 2019^36^	Cross-sectional study	Rural	West	≥ 60	Pune, Maharashtra	135
Pradeep, 2016^32^	Cross-sectional study	Both	South	≥ 16	Krishna, Andhra Pradesh	2934
Gautam, 2012^37^	Cross-sectional study	Both	North	25-55	Sunder Nagar, Himachal Pradesh	300
Rambabu, 2018^38^	Cross-sectional study	Both	South	18-65	Vijayawada, Andhra Pradesh	1155
Sreenivasan, 2015^39^	Cross-sectional study	Both	South	≥ 18	Dharwad, Karnataka	378
Deolia, 2020^40^	Cross-sectional study	Rural	West	≥ 18	Maharashtra	700
Kahar, 2016^41^	Cross-sectional study	Rural	Central	≥ 18	Ramgarh, Madhya Pradesh	35
Gupta, 2015^31^	Cross-sectional study	Rural	West	≥ 20	Western Rajasthan	5476
Kadaluru et al 2012^42^	Cross-sectional study	Urban	South	18-55	Bangalore, Karnataka	246
Kakatkar, 2011^43^	Cross-sectional study	Rural	West	25-45	Udaipur, Rajasthan	427
Basu, 2020^44^	Cross-sectional study	Urban	Central	35-55	NCT Delhi	289
Barman, 2019^45^	Cross-sectional study	Rural	East	18-37	Bhubaneswar, Orissa	300
Baskaradoss, 2020^46^	Cross-sectional study	Both	South	18-35	Kerala	450
Bhaskar, 2020^47^	Cross-sectional study	Both	South	20-59	Kottayam, Kerala	400
Nija, 2020^48^	Cross-sectional study	Both	South	18-34	Kochi, Kerala	194
Khoisnam, 2022^49^	Cross-sectional study	Urban	Central	20-59	Lucknow, Uttar Pradesh	500
Bhuvaneshwari, 2022^50^	Cross-sectional study	Both	South	65-74	Devanagari, Karnataka	1440
Fotedar, 2012^51^	Cross-sectional study	Both	North	25-45	Shimla, Himachal Pradesh	304

###  Quality assessment 

**Figure 2:**
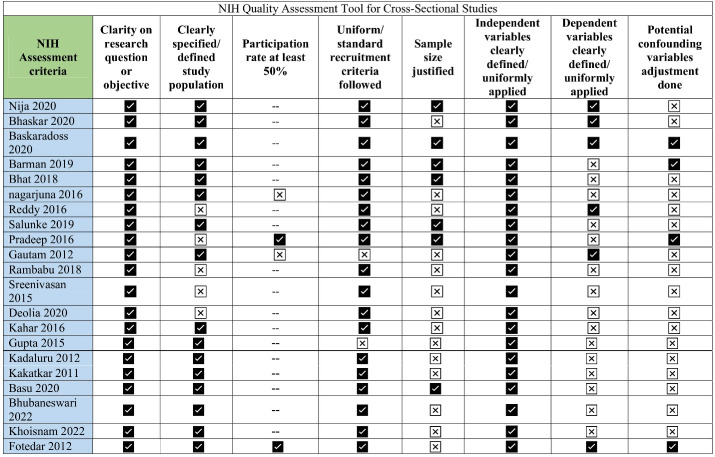
Quality Assessment of studies included in the review

 NIH quality assessment tools for cross-sectional studies were used in this review. Studies were assessed based on eight criteria relevant to the conduction of cross-sectional studies. The median score found for 21 studies was 63.5 with IQR being 73.2 (Q3)–50 (Q1). The complete assessment has been given in Figure 2.

###  Dental care utilization

 The overall pooled estimate of dental care utilization in Indian adult population was estimated as 23.96% (CI: 16.81% - 31.11%), with significant heterogeneity (*P* < 0.001, I^2^ = 98.93%). Moreover, stratified analysis by study zone and study conduction year was performed.

**Figure 3:**
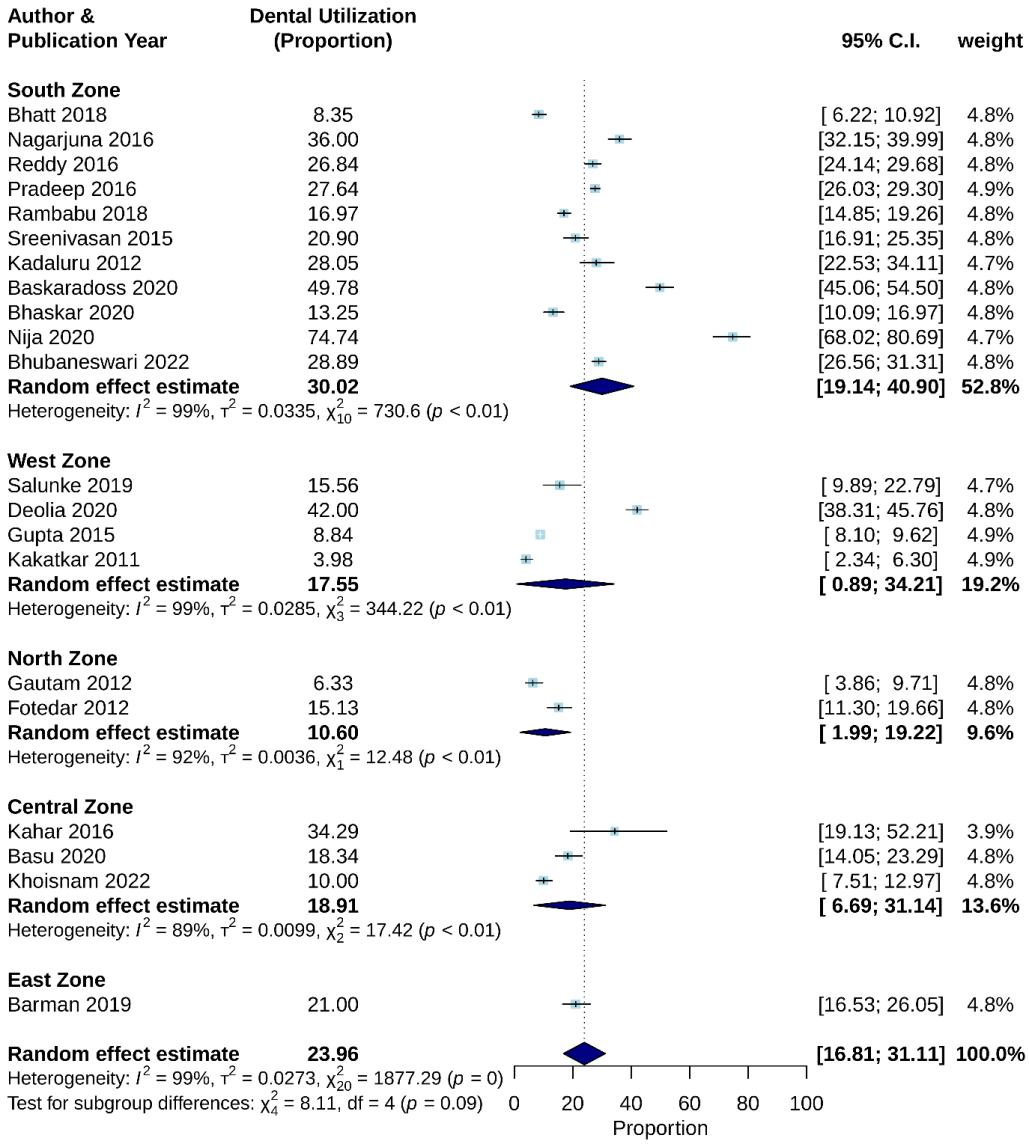
Dental care utilization based on geographic zones

 Based on geographic location, studies from the south zone in India reported a pooled estimate of 30.02% utilization with CI 19.14%–40.90%, with I^2^ value of 98.63% and *P* < 0.01. Pooled sub-estimates of other geographic zones have been depicted in Figure 3.

**Figure 4:**
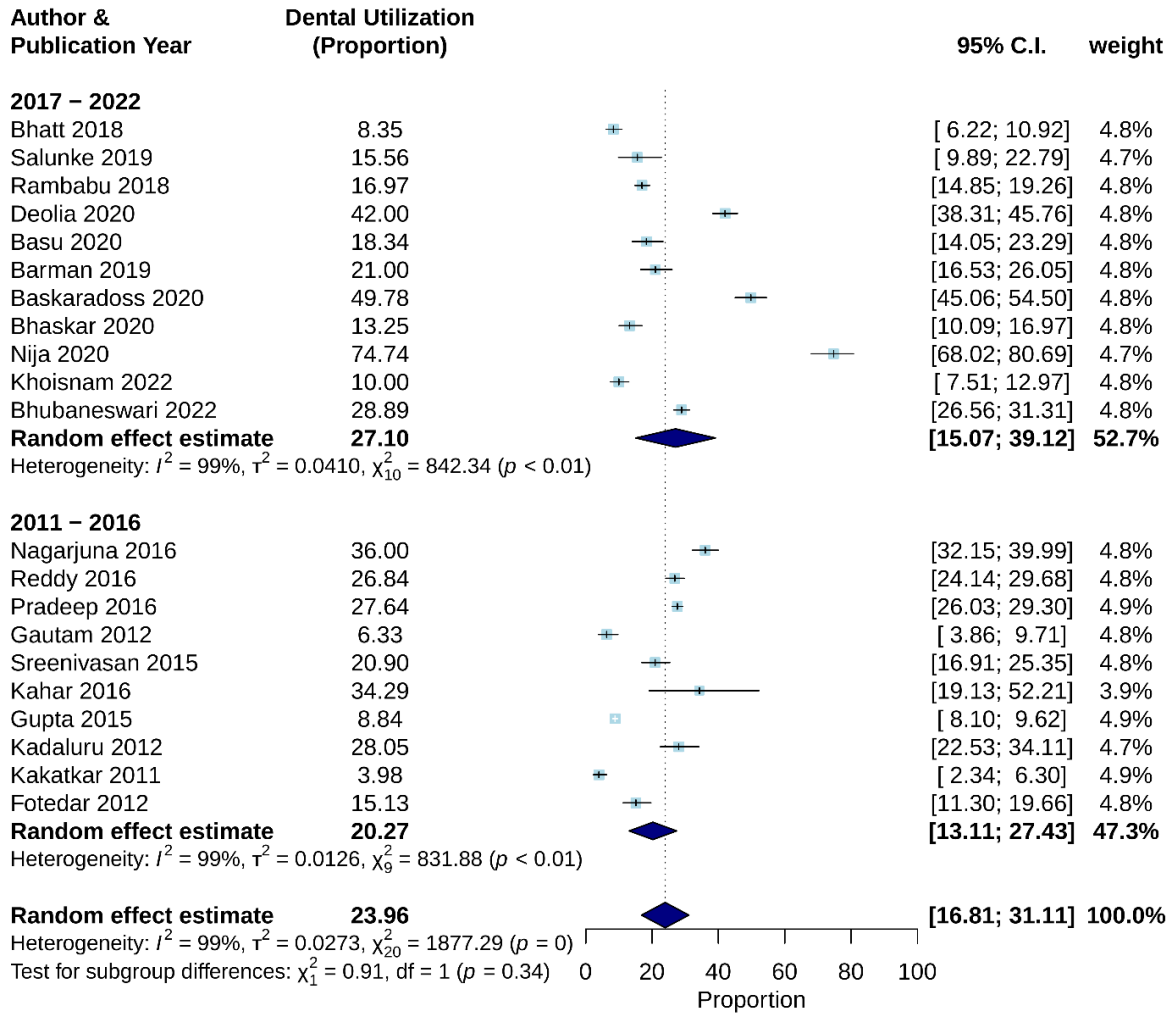
Dental care utilization based on year of study

 Though not statistically significant, when studies were sub-grouped in terms of publishing year a 6.83% increase in utilization was observed in studies after 2017 (*P* = 0.34). Studies published in the last 5 years (2017–2022) yielded a pooled utilization estimate of 27.10 (CI: 15.07–39.12%) with I^2^ = 98.91, *P* < 0.01 (Figure 4).

 According to the test for subgroup differences, there is no statistically significant subgroup effect for variable study zones and study conduction year, with *P* values reported as 0.09 and 0.34, respectively (Figures 3 and 4). However, an uneven distribution of studies contributed to the different study zones in this review, therefore the analysis may not be able to identify differences between subgroups.

 Amongst studies which enrolled only female participants revealed a pooled utilization of 33.67% (CI: 9.39%–57.96%, I^2^ = 99%, *P* ≤ 0.01). Further, a pooled utilization estimates of 19.53% (CI: 9.43–29.63%, I^2^ = 98.4, *P* < 0.01) was found in seven studies that enrolled participants from rural settings only, whereas from five studies that recruited participants from urban setup pooled utilization was 18.15% (CI: 10.10–26.21%, I^2^ = 97.2%, *P* < 0.01).

 The median utilization in terms of proportion recorded from 21 studies was 20.89% with IQR 31.6–11.6%. The study by Nija et al^48^ was identified as an outlier with utilization being 3 standard deviations away from mean utilization. Thereby, sensitivity analysis was performed by removing the study with the highest estimates of utilization reported. The pooled utilization had a minor drop to 21.33% with a negligible drop in heterogeneity (I^2^ = 98.74%, *P* ≤ 0.001). Sensitivity analysis inferred that the pooled estimate did not suffer any major changes thereby establishing the reliability of our pooled estimate.

 Univariate meta-regression analysis revealed no significant association of study variables (study zones, study settings, year of study, participants gender (female/both)) with the pooled estimate of proportions. There were 5 studies that enrolled only female participants.^45-49^ We have tried to assess the possible effect of these studies on overall pooled estimates. Though statistically non-significant a very weak association was found (R^2^ = 6.13%, *P* = 0.12) in moderating the overall pooled estimate. Multivariate regression analysis was not further performed as no variables in question could be found significant in explaining the heterogeneity through univariate analysis.

###  Publication bias

**Figure 5:**
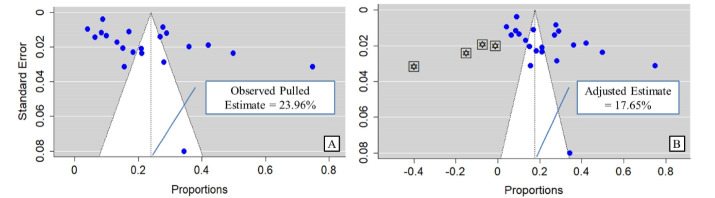
A. Funnel plot depicting publication bias; B. adjusted pooled estimate with trim n fill analysis

 The funnel plot’s asymmetry was observed visually, and to further evaluate any potential publication bias in the literature, the Egger’s test and Begg’s test were also run in this meta-analysis. The Egger’s test and Begg’s funnel plot’s shapes demonstrated that there was evidence of publication bias (*P *value for Egger’s = 0.02; *P *value for Begg’s = 0.04). In order to adjust for publication bias while calculating overall dental utilization among the Indian population, the Duval and Tweedie “trim and fill” method was used. After adjusting for publication bias the pooled estimate had a minor drop to 17.5% from 23.96% with a CI of 8.97–26.33% (*P*= 0.03) **(**Figure 5). We retain our unadjusted pooled utilization estimate as it (23.96%) falls within the confidence limit of the above adjusted estimate.

## Discussion

 The overall utilization of dental services reported in the present study was 23.96%. Our pooled estimates fared well against reported utilizations in some high-income countries. A cross-sectional study using national health survey data in Saudi Arabia reported a 20% utilization by adults.^52^ Similarly, China has reported utilization of 24.28%.^53^ Utilization amongst European developing countries was reported as 20%.^54^ Availability of health insurance schemes was identified as a factor which uplifted the dental service utilization levels at 92.9% in the US as reported in a study conducted by Lutfiyya et al.^55^

###  Zone-wise utilization of the dental services

 Zone-wise comparisons were made and the highest levels of dental service utilization were noted in the southern zone (30.02%) followed by the western zone (17.55%). The availability of the studies reporting the utilization from the East & North-Eastern zones was almost negligible. The reasons for uneven utilization of dental services throughout India could be attributed to the unequal distribution of the 323 dental colleges throughout the country. The southern zone including Karnataka and Tamil Nadu harbors the majority of the dental colleges.^56^ Sparsely distributed dental colleges in the eastern and north-eastern zone rationalize the minimal reporting of the utilization of the dental services among people residing in these zones. Another noteworthy fact about dental care in India is that, although the country now has the needed dentist population ratio, it did so at the expense of significant privatization of dental practice and education. Together, these problems have increased the expense of dental health care.^57^ Further identified barriers to the utilization of the dental services also include the accessibility and affordability of the dental services, the socio-economic status of the residents, literacy rates along with their knowledge and attitude towards availing dental services.^43^ As per the National Family Health Survey (2019-2021), the literacy rates of the southern states were comparatively higher with Kerela at 96.2, Tamil Nadu at 82.9, and Karnataka at 77.2 as compared to the other parts of the country.^58^ Multiple authors have reported a correlation between literacy levels and oral health service utilizations (OHSU). The concept of oral health literacy (OHL) has emerged in the last two decades and has included knowledge related to oral health practices and well-being. Several studies have reported a positive association between the OHL and OHSU.^46,59,60^ Recommendations to conduct quality studies pan-India could improve the research database along with improving the levels of dental care utilization.

###  Time-wise utilization of the dental services

 The dental utilization between 2011 and 2016 was found to be 20.27% in the present meta-analysis. This was seen to increase to 27.10% between 2017 and 2022. These findings can be compared with the results found through the World Health Survey conducted in six states of India dated back to 2003. Amongst the respondents, 28% had oral health issues whereas respondents who availed dental treatment was in the range of 21 to 28%.^61^ Improvements in dental utilization could be rooted in to increase in the awareness among the dental fraternity towards educating the general population about the benefits of maintaining oral health, incorporating mandatory out-reach programs in the dental curriculum, conducting “Reaching the unreached” - theme-based conferences have made way to multiple innovational quotients in the last five years.^62^ The acceptance of overall oral health improvement projects by several national and international funding agencies have led to a drastic shift towards improving the available dental care services among a larger count of people. Ministerial efforts along with the World Health Organization (WHO) funding have introduced the NOHP in 2014-2015 for improving the overall health access and utility of different sections of the Indian population. They have mandated the availability of dental surgeons in government hospitals along with promoting school oral health programs to educate different sections of the population. National Oral health policy has finally found a space in the National Health Policy 2021 setting several targets for improving OHSU.^63^ Rashtriya Bal Swasthya Karyakram (RBSK) has also included dental screening tools as a part of regular medical checkups; thereby improving the utility of dental health care.^64^ Some private health insurance schemes have also been introduced which have encouraged the utilization of dental services among the Indian population.^65^

 Pooled data estimates from five studies have shown a higher utilization of dental services among women (pooled utilization of 33.67% as reported by this meta-analysis). Factors leading to increased utilization included a history of pregnancy, lactating status, levels of awareness, and the willingness to report the minor dental problem as compared to males.^46^ Literature reports have established that pregnancy is characterized by incidences of gingival and periodontal illness.^45^ Inadequate oral hygiene maintenance, systemic co-morbidities, poor awareness about the need for dental care, and failure to seek routine dental care during antenatal visits have been identified as reasons for poor oral health conditions affecting dental care utilization.^66^

###  Strength and Limitations 

 To the best of our knowledge, this is the first study of its kind to pull a national level estimate of dental care service utilisation among adult Indian population from existing local and regional data. One of our study’s limitations is the variance in sampling frame and recruited study population among the primary included studies, which may restrict generalizability. However, despite the observed variability, the resulting figures provide an insight of the demand side of oral health care in India across various Indian regions. Further an evidential lacuna could be detected which necessitates more regionally representative research especially from East and North-eastern zones of India. More studies exploring oral health care utilization, service accessibility and adequacy are needed in different parts of the country. Recently the WHO has published first of its series on “Global Oral Health Status Report” and has envisaged inclusion of oral health interventions under UHC by 2030.^67^ Real-world evidences on the magnitude of the issue, the need and demand for a solution, the cost of interventions, and their effectiveness are essential for any policy reform to occur. This review attempts to address one aspect of the evidence and urges for further future research on all of the aforementioned areas.

 Though we searched PubMed, DOAJ, ScienceDirect, and Google Scholar systematically and meticulously, we acknowledge that we may have missed a few prospective studies that could be found in other digital databases. Another limitation of our review was that we could not compare utilisation across different socioeconomic statuses, gender, and study settings due to the unavailability of variable-specific stratified data.

## Conclusion

 The overall dental utilization in India was found to be around 24%, with South India having the highest usage at 30%. These findings are in objective agreement with variances in the performance of the general health system and variations in the literacy rate, among other factors. Additionally, there was a clear variation in the volume and quality of dental research conducted across India. The databases used for the search contain very few studies from India’s northeast that are relevant to our goal. Studies from the West and East were likewise limited. Incorporating dental insurance into the country’s current public health insurance policies and stepping up oral health promotion initiatives could both serve to enhance comprehensive oral health and health care services at the individual and societal levels.

## Author Contributions

**Conceptualization:** Rounik Talukdar, Diplina Barman, Suman Kanungo.

**Data curation: **Suman Kanungo, Rounik Talukdar, Vallabh Thakkar.

**Formal Analysis: **Rounik Talukdar, Diplina Barman.

**Investigation:** Vallabh Thakkar, Diplina Barman.

**Methodology: **Rounik Talukdar, Vallabh Thakkar, Diplina Barman.

**Project administration: **Suman Kanungo, Rounik Talukdar.

**Resources: **Suman Kanungo.

**Software: **Rounik Talukdar, Diplina Barman.

**Supervision: **Suman Kanungo.

**Validation: **Vallabh Thakkar, Diplina Barman, Rounik Talukdar.

**Visualization:** Rounik Talukdar, Diplina Barman.

**Writing – original draft: **Rounik Talukdar, Diplina Barman.

**Writing – review & editing: **Suman Kanungo, Diplina Barman, Vallabh Thakkar.

## Funding

 Nil.

## Ethical Approval

 Not applicable.

## Competing Interests

 The authors have no conflicts of interest to disclose.

## Supplementary File


Supplementary file 1 contain PRISMA check list.
